# A High-Performance System for Weak ECG Real-Time Detection

**DOI:** 10.3390/s24041088

**Published:** 2024-02-07

**Authors:** Kun Xu, Yi Yang, Yu Li, Yahui Zhang, Limin Zhang

**Affiliations:** 1 School of Electronic Science and Engineering, Nanjing University, Nanjing 210023, China; kxu@smail.nju.edu.cn (K.X.); mf20230136@smail.nju.edu.cn (Y.Y.); mf21230061@smail.nju.edu.cn (Y.L.); mf21230166@smail.nju.edu.cn (Y.Z.); 2 The Life and Health Industry Research Institute of Nanjing University, Zhenjiang 212000, China

**Keywords:** ECG, noise suppression, multi-channel system, electrode array

## Abstract

Wearable devices have been widely used for the home monitoring of physical activities and healthcare conditions, among which ambulatory electrocardiogram (ECG) stands out for the diagnostic cardiovascular information it contains. Continuous and unobtrusive sensing often requires the integration of wearable sensors to existing devices such as watches, armband, headphones, etc.; nonetheless, it is difficult to detect high-quality ECG due to the nature of low signal amplitude at these areas. In this paper, a high-performance system with multi-channel signal superposition for weak ECG real-time detection is proposed. Firstly, theoretical analysis and simulation is performed to demonstrate the effectiveness of this system design. The detection system, including electrode array, acquisition board, and the application (APP), is then developed and the electrical characteristics are measured. A common mode rejection ratio (CMRR) of up to 100 dB and input inferred voltage noise below 1 μV are realized. Finally, the technique is implemented in form of ear-worn and armband devices, achieving an SNR over 20 dB. Results are also compared with the simultaneous recording of standard lead I ECG. The correlation between the heart rates derived from experimental and standard signals is higher than 0.99, showing the feasibility of the proposed technique.

## 1. Introduction

Cardiovascular disease is one of the major health problems and the leading cause of death in the world [1,2]. With the development of modern society, wearable devices have been widely used in home medical monitoring and can be applied in various physiological parameters and scenarios of health conditions [3,4]. ECG device is helpful to monitor cardiovascular diseases and holds a pivotal position in home medical care [5,6]. 

ECG signal is a low-frequency, low-amplitude signal with an amplitude varying between 0.01~4 mV and a frequency between 0.05~150 Hz, which could be easily contaminated by various interference, such as power-line noise, electromyography (EMG), etc. [7]. Signal processing algorithms for noise suppression are proposed to solve the problem, such as notch filter [8], wavelet transform [9], empirical mode decomposition (EMD) [10], and adaptive filtering [11]. With the rapid development of the neural network technology in recent years, artificial neutral networks (ANN) have been used in the reconstruction of high-quality ECG signals [12]. In addition to the above techniques, efforts have also been made to optimize hardware circuits [13]. To reduce the influence of the error of the peripheral components on CMRR, a novel front for bioelectrical signal acquisition is proposed [14]. The detection electrodes are very susceptible to the power line common mode interference (CMI) and the body motion. Y. Tang propose a noise neutralization method by applying the reference electrode and a 50 Hz band-pass filter to obtain the interference of the human body and adapting the gains to neutralize the interference inputs of two acquisition electrodes and achieve the minimum interference output [15]. Meanwhile, an electrode-controllable humidification design using ultrasonic atomization is proposed to suppress motion artifacts [16].

Existing wearable ECG monitoring is commonly realized by ECG patches attached to the chest, which will make the user feel uncomfortable and are inconvenient for long term monitoring. In contrast, other ECG devices worn on the arm, wrist, and ear are likely more acceptable [17]. ECG can also be acquired from the wrist through a wristband or watch by actively attaching the idle arm to the device [18,19]; however, it impedes the continuous cardiovascular monitoring provided by ECG patches and can only provide episodes of recordings for diagnosis. Meanwhile, signals from the arm and ear are low in amplitude (in scale of microvolts), on one hand ownig to the relatively large distance from the heart, and on the other hand, because of the short distance of the two differential electrodes, which increases the difficulty of detecting high-quality ECG signals [20,21]. Table 1 shows the amplitude of ECG acquired from different locations on the body: for example, the typical amplitude of ECG signal from the chest is 1 mV; however, the amplitude from mastoid area is only about 30–50 μV, which is 3–5% the amplitude of the chest after neck attenuation [22]. The test results from references [23,24] show a peak-to-peak amplitude of 30–50 μV and are not complete ECG signals, which leads to difficulty for continuous ECG monitoring. Jacob N K et al. attempted to conduct ECG measurements behind one ear, but only limited information (R wave) could be observed [25]. Because of the periodicity of ECG signal and the waveforms in each cycle are roughly the same, efforts have been carried out in Average Evoked Response (AEV) to remove noise in ECG. However, the key to using the AEV method is to find the superimposed time reference point, which should not be contaminated by noise, hindering the real-time monitoring of ECG [26]. A method for suppressing synchronous carrier wave interference (CWI) based on accumulation and average is presented according to the characteristics of Loran-C pulses structure with aligning signals from different channels to eliminate phase difference [27].

The aim of this paper is to propose a multi-channel device using noise suppression method for weak ECG real-time detection. The outstanding characteristic of the proposed device is applying multi-channel signal superposition to suppress the interference in real time without complex signal processing. The paper is organized as follows. Firstly, the theoretical analysis and simulation of the proposed method are given to show the improved performance. Then, the system with multi-channel signal detection is introduced, including the design and the electrical performance. Finally, ECG detection cases behind the ear and from the upper arm are given to verify the feasibility of the proposed method. 

## 2. Theoretical Analysis

ECG signal is usually contaminated with three kinds of noises: the baseline wander, powerline interference, and electromyogram (EMG) [28], which can be reduced by related algorithms. To detect weak ECG signal, the short-circuit input noise of the detection system should be as small as possible. The system noise usually comes from the detection chips, which can be seen as random and unrelated to the ECG signal. Therefore, the collected signal can be expressed as a linear sum of ECG signal and unrelated noise.

(1) $$g\left(t\right)=f\left(t\right)+\eta \left(t\right)$$

where $f\left(t\right)$ is the ECG signal and $\eta \left(t\right)$ is the unrelated noise. If the detection electrodes of a multi-channel system are placed in proximity such that the detected ECG signal from each channel can be seen as the same, the averaged signal detected by multi-channel system can be expressed as follows:

(2) $$\bar{g}\left(t\right)=\frac{1}{K}[\sum_{i=1}^{K}f_{i}(t)+\sum_{i=1}^{K}\eta_{i}\left(t\right)]=f\left(t\right)+\frac{1}{K}\sum_{i=1}^{K}\eta_{i}\left(t\right)$$

where *K* is the total number of channels, $f_{i}\left(t\right)$ is the ECG signal of channel *i* and can be assumed to be the same as $f\left(t\right)$, and $\eta_{i}\left(t\right)$ is the unrelated noise of channel *i*.

Since the noise is random and unrelated interference, the expectation of the averaged signal can be obtained as follows:

(3) $$\begin{array}{c} E\left[\bar{g}\left(t\right)\right]=f\left(t\right) \end{array}$$

Variance of the averaged signal is as follows:

(4) $$\begin{array}{c} \sigma_{\bar{g}\left(t\right)}^{2}=\frac{1}{K}\sigma_{\eta \left(t\right)}^{2} \end{array}$$

(5) $$\begin{array}{c} \sigma_{\bar{g}\left(t\right)}=\frac{1}{\sqrt{K}}\sigma_{\eta \left(t\right)} \end{array}$$

It can be seen from Equations (3)–(5) that the averaged signal measured by a multi-channel system is expected to be a pure ECG signal with a $\sqrt{K}$ part of unrelated noise, which means the signal–noise ratio (SNR) can be improved by $\sqrt{K}$ times. In the case of eight channels, SNR can theoretically be improved by $\sqrt{8}$ times, that is, about 9.03 dB.

**Figure 1:**
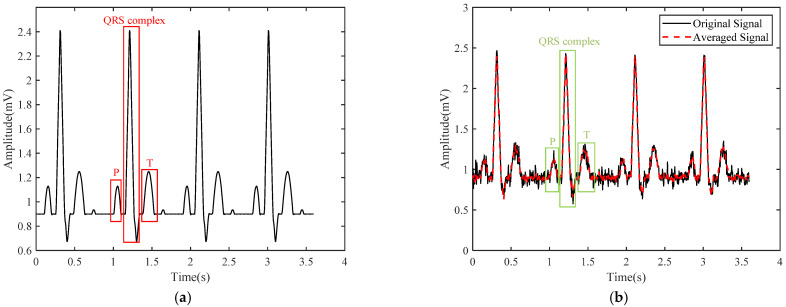
(**a**) Standard ECG waveforms. (**b**) ECG waveforms disturbed with noise and the waveform averaged with eight channels.

Figure 1a shows a standard ECG waveform with four cycles generated by computer simulation with 180 data points in one ECG cycle, where the P wave, QRS complex, and T wave are clearly visible. If a Gaussian noise is added to the standard ECG waveform, the P wave and T wave of each channel will be disturbed and indistinguishable, as shown with the dashed line in Figure 1b. If eight channels are contaminated by noise together, the averaged signal calculated with Equation (2) is also shown with the solid line in Figure 1b, where the noise is obviously suppressed. The SNR can be calculated by Equation (6), where (QRS) *ECG_p-p_* is the peak-to-peak amplitude of the R wave in a 120 ms span centered with the R wave and (T-P) *noise_p-p_* is the peak-to-peak amplitude of noise in a 40 ms R-T time interval span [29]. After calculation, the SNR can be improved by up to 10 dB.

(6) $$SNR=20log\frac{\left(QRS\right){ECG}_{p-p}}{\left(T-P\right){noise}_{p-p}}$$

**Figure 2:**
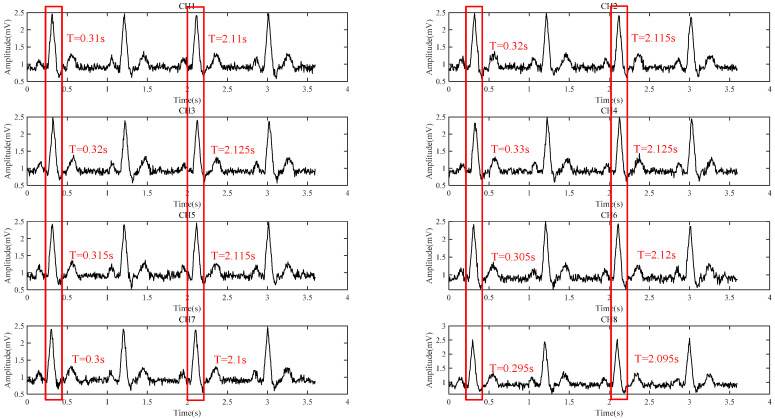
Eight-channel ECG waveforms with noise and phase difference.

It should be pointed out that the detected ECG signal from each channel may be vary slightly in phase if the electrodes are not placed close enough together. If there is a point difference between different channels, 2° difference will be generated in phase. In Figure 2, the eight-channel ECG signal is slightly phase shifted, with a phase difference of 2° between each channel, and it can be seen that the arrival time of the R peak between each channel is not exactly the same. In this case, the simulation results of the averaged signal by Equation (2) show that SNR is only improved about 2–3 dB, which is not as ideal as the case without phase difference between each channel shown in Figure 2. In an actual test, there may also be phase misalignment between each channel of the multi-channel system; therefore, the key to the success of the proposed method is to make the signal of each channel as similar as possible.

## 3. System Architecture

**Figure 3:**
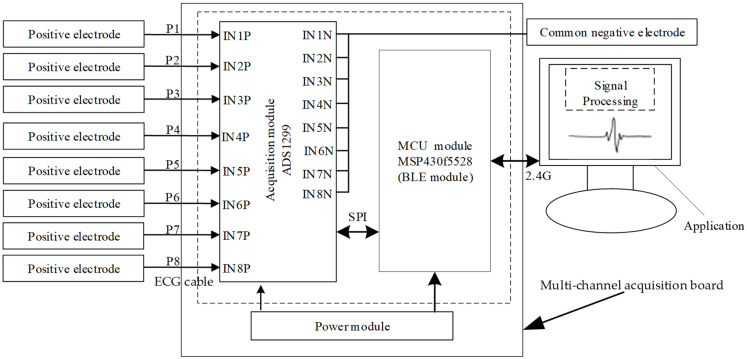
System block diagram.

The whole detection system includes the following parts: electrode array, acquisition board, and the APP, as shown in Figure 3. The electrode array is attached to the human body to collect the ECG signals, and the ECG signals are processed by the acquisition board and visualized in real time through the APP.

### 3.1. Electrode Array

**Figure 4:**
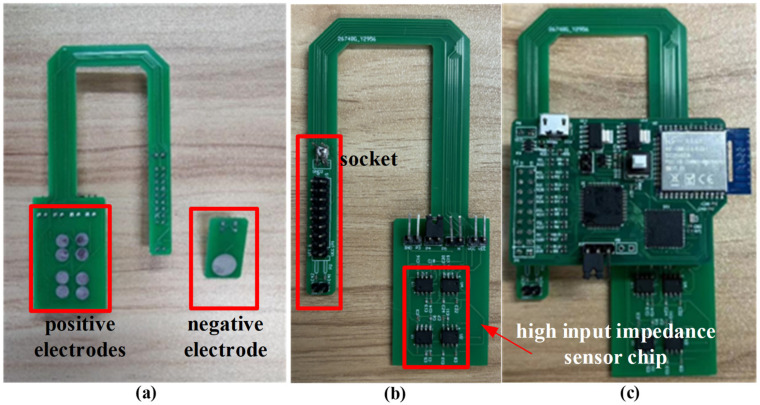
Eight-channel electrode array for behind-ear ECG test: (**a**) back view, (**b**) front view, (**c**) with acquisition board.

**Figure 5:**
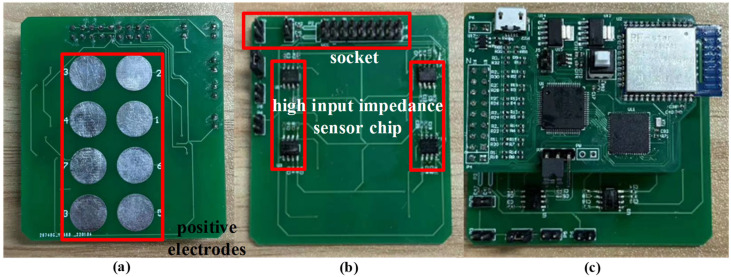
Eight-channel electrode array for upper arm ECG test: (**a**) back view, (**b**) front view, (**c**) with acquisition board.

In order to ensure the signal of each channel is as same as possible, an electrode array with eight positive electrodes and one negative electrode is used to collect the ECG signals. Hook-shaped and rectangle-shaped electrode arrays are designed for ECG tests behind the ear and on the upper arm, respectively, as shown in Figure 4 and Figure 5, where each electrode is a circular plate with a diameter of 5 to 10 mm and tin plating on its surface, and the distance between adjacent electrodes is 3 to 5 mm. 

For the behind-the-ear ECG test, the diameter of each electrode is 5 mm and the size of the electrode array is 24 × 13 mm, and the hook shape enable the board to be hung from the ear, allowing the electrodes to make better contact with the skin. If the electrode diameter is too small, the signal cannot be collected well; otherwise, the electrode area will be too large and affect the wearing comfort. For the area behind the ear, the electrode diameter of 5 mm is a relatively appropriate size. Meanwhile, it takes time for ECG signals to propagate from the heart to the body surface, which results in the signals collected at different locations on the human body not arriving at the same time, so we make the spacing between electrodes as small as possible to ensure that the signal of each channel is as similar as possible. For the upper arm ECG test, the diameter of each electrode is 10 mm and the size of the electrode array is 50 × 26 mm. The space here is larger than that behind the ear, so the 10 mm electrode diameter is adopted to ensure the quality of signal acquisition. Compared with the medical Ag/AgCl electrode, the electrode is dry when using tin surface detection and does not easily produce allergic reactions on the skin, which can meet the requirements of long-term ECG monitoring. Each electrode is connected with an ultra-high input impedance sensor chip located on the electrode back [30]. The chip is characterized by an input capacitance of about 10 pF, an input impedance of about 150 GΩ, and a short-circuit noise of about 0.9 μV, which can efficiently sense the charge signal on the electrode and output it to the acquisition board.

### 3.2. Acquisition Board

As shown in Figure 4, the multi-channel ECG acquisition board consists of three parts: power module, acquisition module, and MCU module. The power module supplies the system by a rechargeable lithium polymer battery. TPS73801, a low dropout regulator with noise below 45 μV_RMS_ (10 Hz to 100 kHz), is used to convert the 3.7 V voltage of the battery to 3.3 V to drive the digital circuits. The acquisition module is in charge of collecting the signal from the electrode array, and the integrated front-end for bioelectricity measurement chip, ADS1299, is used to implement the function, which has eight differential input channels with 24 bit ADC and a programmable gain amplifier (PGA) with gain between 1 to 24. At the same time, ADS1299 supports programmable sampling rate configuration from 250–4k SPS. High precision ADC makes the voltage resolution of the system very high, enabling the acquisition of weak ECG signals without a high gain amplifier. Eight positive electrodes connect to the P input end of the ADC chip in sequence according to the channel order and one negative electrode connects to the N input end of the ADC chip for all eight channels. A first-order high pass filter with a characteristic frequency of 0.15 Hz is placed in front of the input channels to filter out the baseline drift noise, where 1% accuracy resistance or capacitance elements of the filter are selected to ensure the phase differences caused by parameter errors of filters within 2°. The equal length differential lines in the PCB of the acquisition board are also used to further ensure signal synchronization. The gain of each channel is set as 12 to ensure amplifying the ECG signal to the input range of the ADC input. The sampling rate of each channel is set as 250 Hz to meet the frequency band from 0.1 Hz to 70 Hz of the ECG signal. 

**Figure 6:**
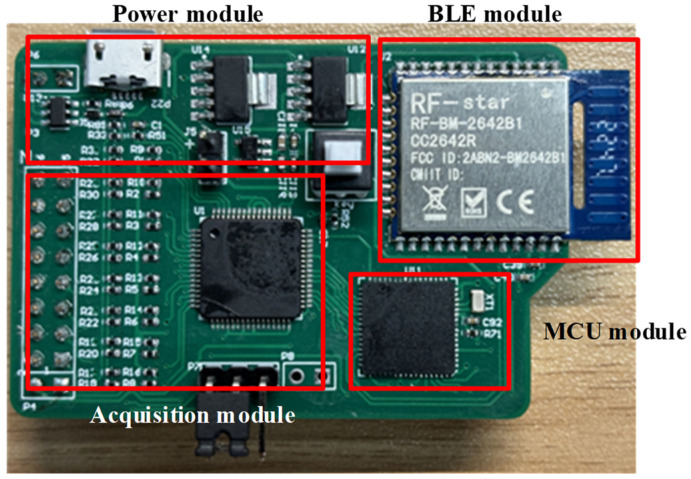
Developed acquisition board.

The chip MSP430F5528 is selected as the MCU module; its built-in serial communication port can transmit collected data from acquisition module to the application. The eight-channel 24 bit data is sent to the MCU every 4 ms, and a 26 byte data packet is formed by adding the header and tail inside, which is sent out through the serial port module. The baud rate of serial communication is set to 115,200 bps, which can ensure real-time transmission of data for eight channels at the sampling rate of 250 Hz. The developed acquisition board is shown in Figure 6 with a size of 51 × 38 mm.

### 3.3. The Application

**Figure 7:**
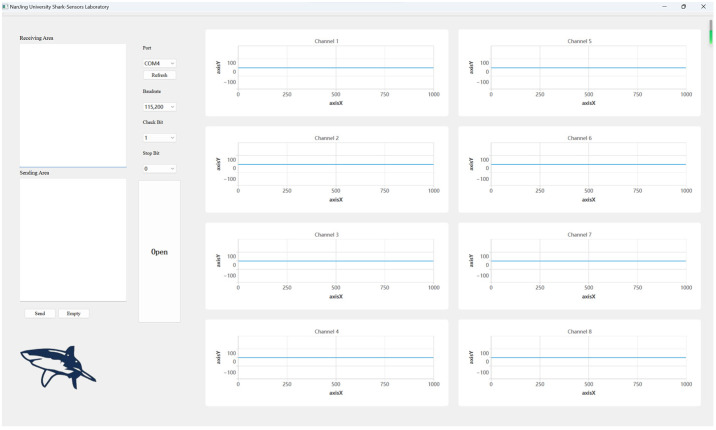
Window of the application.

The function of the APP is to display the collected signals from the acquisition board and achieve the real-time data processing. The APP is developed with Qt 6.0 software, which is divided into eight areas to show the corresponding channel signal, as shown in Figure 7. Meanwhile, data processing is carried out in real time to remove the interference, including the notch of 50 Hz and 100 Hz, low-pass filter of 100 Hz, and high-pass filter of 0.1 Hz. The phase problem from filtering is solved by the method of first backward filtering and then forward filtering to achieve zero phase-shift effect.

### 3.4. Electrical Characteristics

The common mode rejection ratio (CMRR) is an important index to measure the ability of the system to suppress common mode interference, which is defined with the ratio of the gain of differential input signal to common input signal. Here, CMRR is measured with the common input 50 Hz sinusoidal signal of the amplitude 1000 mV and the differential input 50 Hz sinusoidal signal of the amplitude 100 mV. Considering that the performances of the eight channels are almost accordant, Table 2 shows the measured results of one channel, where CMRR is higher than 100 dB.

**Figure 8:**
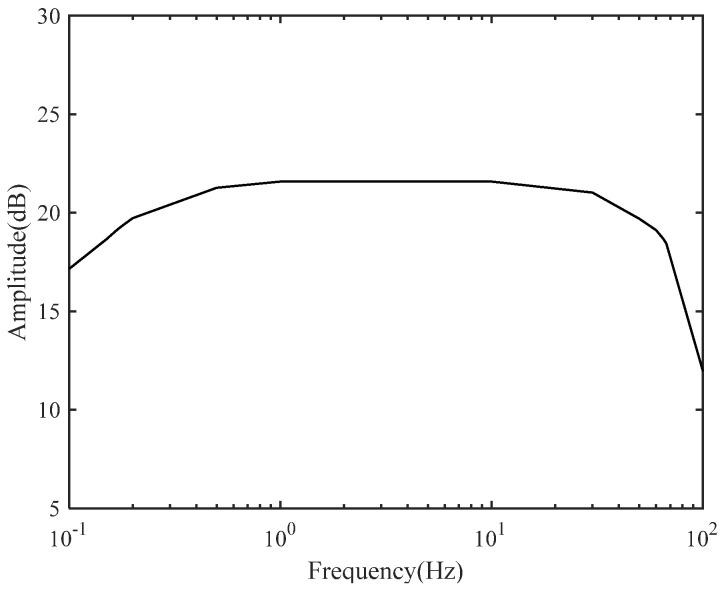
Frequency response measurement result.

By introducing the 100 mV sine wave signal generated by the signal source as the test signal, the amplitude frequency response curve of the system can be obtained with the gain of 21.76 dB and bandwidth from 0.18 Hz to 66.0 Hz, as shown in Figure 8.

When the input terminals are short-circuited to the ground, the input reference voltage noise can be measured. As shown in Table 3, the input referred voltage noise of each channel is distributed between 1.2 μV and 1.7 μV. Afterwards, the noise of eight channels is superimposed with Equation (2) and the averaged noise can be reduced to 0.65 μV.

According to the National standards YY1079-2008 [31] in the medical industry, the short-circuit noise of electrocardiogram monitors should not exceed 30 μV, and the CMRR for 50 Hz interference should be larger than 80 dB, so the electrical characteristics of the proposed system can fully meet the low noise requirement of weak ECG measurement.

## 4. Results and Analysis

To verify the feasibility of the proposed multi-channel noise suppression method, the ECG signals from behind the ear and on the upper left arm are measured, and the results are compared with the existing methods.

### 4.1. Ear-Behind ECG Test

**Figure 9:**
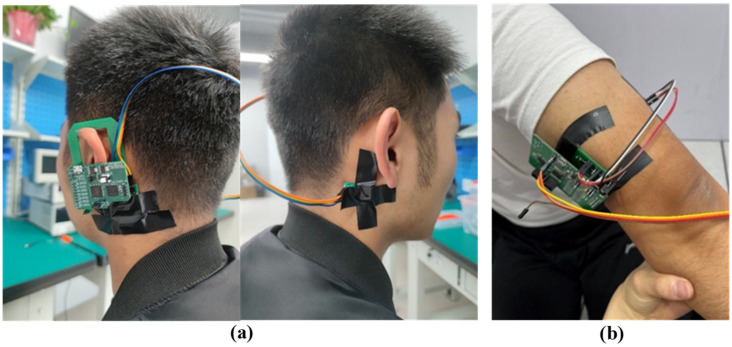
ECG test: (**a**) behind the ear, (**b**) upper arm.

To imitate the position of an earphone in the ear, the electrodes are placed at the mastoid, with the electrode array placed behind the left ear and the common negative electrode placed behind the right ear, where the wires between the two ears are used for power supply and signal transmission, as shown in Figure 9a. During the testing period, the experimenter remains stationary, and the experiment lasts for 2 min. 

**Figure 10:**
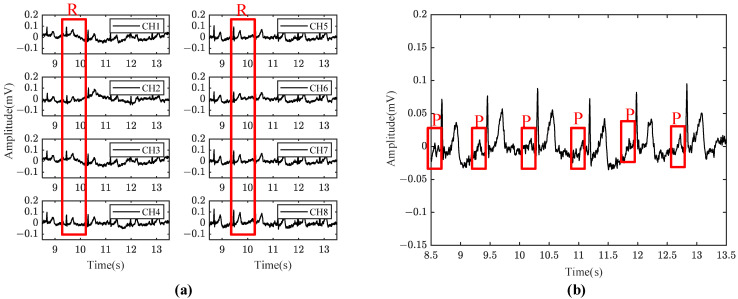
(**a**) Eight-channel behind-the-ear ECG waveforms; (**b**) behind-the-ear ECG eight-channel average waveform.

**Figure 11:**
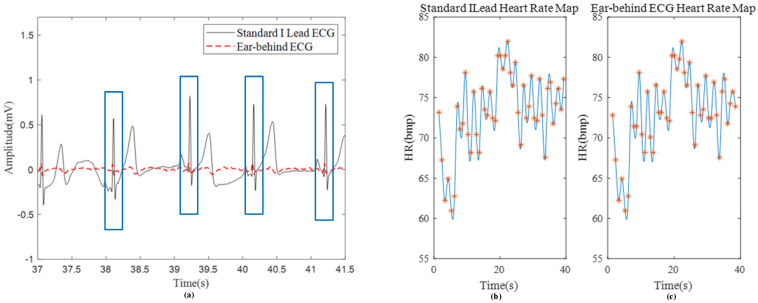
(**a**) Comparison of behind-the-ear ECG with standard lead ECG; (**b**) heart rate diagrams of standard I lead; (**c**) heart rate diagrams of behind-the-ear lead.

The ECG signal behind the ear is shown in Figure 10a, where CH1 to CH8 represent the signals of channel 1 to channel 8 of the system, respectively. The averaged signal represents the averaged signal of eight channels of the system, as shown in Figure 10b. It can be seen that the R peak amplitude of the averaged ECG signal is about 100 μV and the SNR of averaged ECG signal is improved to 21.5 dB from 15.1 dB of single ECG signal in Figure 10. The P wave of the ECG signal in Figure 10b is clearly visible. Figure 11 shows the comparison between the ECG signals measured behind the ear and the standard I ECG signals measured on both wrists. It can be seen that the R peak of the two ECG signals are basically aligned, indicating that the R-R interval between the two signals is consistent, and the calculated heart rate diagrams show that the correlation between the two heart rates is more than 0.99, indicating that the signals measured by this system are indeed ECG signals. Moreover, this system can completely measure P wave, QRS complex, and T wave signal of ECG, which shows the possibility for heart rate monitoring and disease prevention judgment. As shown in Table 4, the proposed system has been compared with some other state-of-the-art systems, which can only see R-waves for heart rate detection and cannot detect P wave and T wave for other disease diagnosis. However, these achievements did not provide clear SNR values, so a qualitative evaluation based on their signal waveforms is conducted.

### 4.2. Upper Arm ECG Test

In the upper arm test, the electrodes are placed at the position of the biceps of the upper arm, as shown in Figure 9b. The positive electrode array and the common negative electrode are located on the inside and outside of the arm, respectively, in order to keep the distance between the electrodes as far as possible to acquire a better signal. During the testing period, the experimenter remains stationary, and the experiment lasts for 2 min.

**Figure 12:**
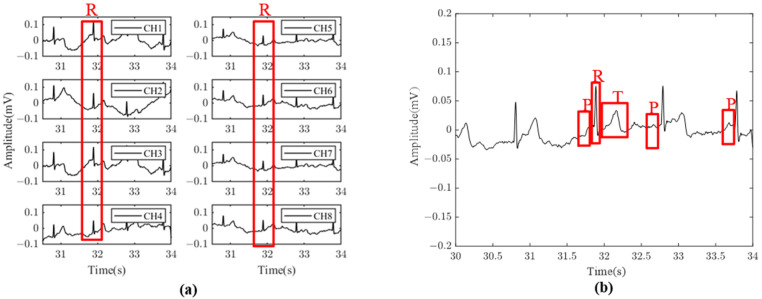
(**a**) Eight-channel upper arm ECG waveforms; (**b**) upper arm ECG eight-channel average waveform.

**Figure 13:**
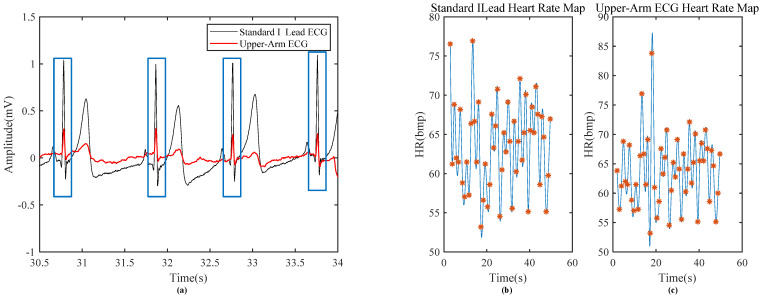
(**a**) Comparison of upper arm ECG with standard lead ECG; (**b**) heart rate diagrams of Standard I lead; (**c**) heart rate diagrams of upper arm lead.

Figure 12 shows the signal acquired from the upper arm, which can clearly distinguish the R and T bands with a maximum amplitude of 70 to 80 μV, and the P wave is sometimes available. The SNR of the single channel is 16.5 dB, and the SNR of the averaged ECG signal is 22.7 dB with an improvement of 6.2 dB, which is concordant with the theoretical analysis. In addition, the comparison between the data measured on the upper arm with the proposed system and the standard lead of the wrist acquired by another ECG equipment designed by our group [14] is also carried out, as shown in Figure 13; each R-peak is manually aligned, indicating that the R-R interval between the two signals is consistent and the heart rate correlation is still above 0.99. As shown in Table 5, the proposed system has been compared with some other state-of-the-art systems, which can only see R wave or T wave for heart rate detection and cannot detect P wave.

## 5. Conclusions

In this paper, a high-performance system with multi-channel signal superposition is proposed to implement weak ECG real-time detection, for example, ECG detection behind the ear and on the upper arm. The outstanding characteristic of the proposed method is applying multi-channel signal superposition to suppress the interference and improve the signal-to-noise ratio. Firstly, the theoretical derivation of multi-channel superposition denoising is described, and the results show that k-channel averaging can suppress the random noise and improve the SNR by $\sqrt{K}$ times. In computer simulation experiments, an eight-channel system can improve the SNR by 10 dB without phase difference between each channel; however, when there is a 2° phase difference in each channel, it can only improve the SNR by 2–3 dB. Secondly, the detection system with eight-channel signal is introduced. The electrical characteristics, including CMRR, short-circuit noise, and frequency response of the system, are measured, and the results show that the performance of the system fully meets the international standards and the weak ECG detection requirements. Finally, ECG detection experiments behind the ear and on the upper arm are carried out, where P wave, QRS complex, and T wave are clearly visible, and the SNR can reach 21 dB and 22 dB, respectively. Moreover, the experimental results are compared with those of the standard I lead at the same time. The correlation between the two heart rates is more than 0.99, which shows that the reliability of the proposed design.

Future work aims to reduce the size of the system, improve the quality of the electrodes, and design new forms of electrodes to better fit the shape of the ear.

## Figures and Tables

**Table 1:** Amplitude of ECG from different locations on the body.

Location	Amplitude/μV	Percentage/%
Chest	1000	100
Mastoid	30–50	3–5
Upper-arm	50–70	5–7

**Table 2:** Common mode rejection ratio test results.

Input	Input Frequency		Output		CMRR
	f/Hz	Vpp/mV	Vpp/mV	Av	
Differential mode	50	100	926.5	9.265	100.6
Common mode		1000	0.086	860,000	

**Table 3:** Input referred voltage noise of each channel.

Channel	Vpp/μV
CH1	1.4
CH2	1.5
CH3	1.7
CH4	1.2
CH5	1.5
CH6	1.25
CH7	1.1
CH8	1.2

**Table 4:** Comparison between our system and other state-of-the-art systems at behind-the-ear location.

System	P Wave	R Wave	T Wave	SNR/Noise	Size
Multi-channel system	Y	Y	Y	21.5 dB	Small
Ear-worn ECG&PPG monitor [22]	N	Y	N	High	Large
An Ear-Worn Vital Signs Monitor [23]	N	Y	N	High	Small
“hearables” earpiece [24]	N	Y	N	Moderate	-
Inkjet-printed electrodes [25]	N	Y	N	High	-

**Table 5:** Comparison between our system and other state-of-the-art systems at the upper arm location.

System	P Wave	R Wave	T Wave	SNR/Noise	Size
Multi-channel system	Y	Y	Y	22.7 dB	Small
LUA wearable device [17]	N	Y	Y	High	Small
Biopac M35 [18]	N	Y	N	Low	-
Single-arm monitor [19]	N	Y	N	Moderate	Large
A pilot clinical study [21]	-	-	-	19.8 dB	-

## Data Availability

Data are contained within the article.
